# A Hospital Pageant

**Published:** 1924-03

**Authors:** 


### A PAGEANT OF SOUTH LONDON.

The whole of the Crystal Palace and its extensive grounds will be given up to King's College Hospital on the three last days of May, when a spirited attempt to raise from £15,000 to £20,000 for this admirable institution will be made. The Pageant itself, which is under the direction of Mr. Patrick Kirwan, who was the Pageant-Master at Arundel last year, will take place on the football ground, and it is estimated that some 3,000 performers will take part. It will begin at 5.30 p.m. on each day and is expected to last for three hours. The districts co-operating in the Pageant are each undertaking the entire financial responsibility for the respective episodes, and, under Mr. Kirwan's control, will make their own arrangements and settle their castes. There will be a few speaking parts.

### The Scenes.

The Pageant is in the form of playlets and is divided into the following scenes :—The Last Stand of Boadicea against the Romans at Beckenham (Camberwell); the Marriage Feast of Tostig the Proud and Goda the daughter of Clape at Kennington (Kennington, Brixton, Stockwell and Clapham); the Foundation of Merton Priory (Wimbledon and Merton); the Rising of the Peasants of Surrey and Kent (Penge, Anerley, Beckenham and Shortlands); the Passing of the Canterbury Pilgrims at St. Thomas a-Watering (Southwark); King Henry V. Returns from Agincourt through Southwark (Bromley, Bickley, Hayes and Keston); King Henry's Widow takes Sanctuary at Bermondsey (Chislehurst and Orpington); Honor Oak receives the Virgin Queen with May Revels (Honor Oak, Forest Hill and Sydenham)—in this scene there is an old Elizabeth playlet, "The Merry Jest" (Lewisham, Hither Green, Catford, Grove Park and Brockley); Alleyne gives a College to Dulwich (Dulwich); With Mr. Pepys at Southwark Fair (Herne Hill, Tulse Hill and Norwood); "The Club" visits Vauxhall Gardens (Balham, Tooting and Streatham). The Finale will be a scene illustrating the History of Medicine.

### The Side-Shows.

It is clear that each locality is doing its very best to make this great effort a success, and many prominent local personalities are interesting themselves actively in the production. These include the Members for Streatham and Lewisham, the Headmaster of Dulwich College, and the Mayors of Camberwell, Lambeth, Wimbledon, Wandsworth, Southwark, Bromley and Lewisham. While the main support is likely to come from South London, it is hoped that many visitors from over the river will go to see the Pageant, and the Hospital has arranged with the railway company for the issue of cheap tickets. It will, indeed, be possible to spend a very pleasant day at the Crystal Palace. There will be numerous side-shows, and on the Thursday and Saturday there will be athletic meetings on the sports ground, and over 1,000 entries are expected each day. In the evening, in addition to dancing, the distinguished Operatic and Dramatic Society of the Stock Exchange will perform in the theatre.

